# N-acetylcysteine prevents HIV gp 120-related damage of human cultured astrocytes: correlation with glutamine synthase dysfunction

**DOI:** 10.1186/1471-2202-8-106

**Published:** 2007-12-06

**Authors:** Valeria Visalli, Carolina Muscoli, Iolanda Sacco, Francesca Sculco, Ernesto Palma, Nicola Costa, Carmela Colica, Domenicantonio Rotiroti, Vincenzo Mollace

**Affiliations:** 1 Department of Pharmacobiological Sciences, Faculty of Pharmacy, University "Magna Graecia", Catanzaro, Italy; 2 San Raffaele Pisana IRCCS, Rome, Italy; 3 Istitute Mondino-Tor Vergata, Rome, Italy; 4 Salus Research Institute, Marinella di Bruzzano, Italy

## Abstract

**Background:**

HIV envelope gp 120 glycoprotein is released during active HIV infection of brain macrophages thereby generating inflammation and oxidative stress which contribute to the development of the AIDS-Dementia Complex (ADC). Gp120 has also been found capable to generate excitotoxic effect on brain tissue via enhancement of glutamatergic neurotransmission, leading to neuronal and astroglial damage, though the mechanism is still to be better understood.

Here we investigated on the effect of N-acetylcysteine (NAC), on gp120-induced damage in human cultured astroglial cells and the possible contribution of gp120-related reacting oxygen species (ROS) in the imbalanced activity of glutamine synthase (GS), the enzyme that metabolizes glutamate into glutamine within astroglial cells playing a neuroprotective role in brain disorders.

**Results:**

Incubation of Lipari human cultured astroglial cells with gp 120 (0.1–10 nM) produced a significant reduction of astroglial cell viability and apoptosis as evaluated by TUNEL reaction and flow cytometric analysis (FACS). This effect was accompanied by lipid peroxidation as detected by means of malondialdehyde assay (MDA). In addition, gp 120 reduced both glutamine concentration in astroglial cell supernatants and GS expression as detected by immunocytochemistry and western blotting analysis. Pre-treatment of cells with NAC (0.5–5 mM), dose-dependently antagonised astroglial apoptotic cell death induced by gp 120, an effect accompanied by significant attenuation of MDA accumulation. Furthermore, both effects were closely associated with a significant recovery of glutamine levels in cell supernatants and by GS expression, thus suggesting that overproduction of free radicals might contribute in gp 120-related dysfunction of GS in astroglial cells.

**Conclusion:**

In conclusion, the present experiments demonstrate that gp 120 is toxic to astroglial cells, an effect accompanied by lipid peroxidation and by altered glutamine release. All the effects of gp120 on astroglial cells were counteracted by NAC thus suggesting a novel and potentially useful approach in the treatment of glutammatergic disorders found in HAD patients.

## Background

HIV infection is still a pandemic disease with more than 30 million people infected today. HIV-positive individuals experience cognitive dysfunction, disordered behaviour and problems with movement and balance which are indicated, at the late stage, as HIV-Associated Dementia (HAD) [1,2]. In fact, neurodegenerative disorders have commonly been described in patients suffering from AIDS and this occurs even though neurons are not infected following HIV-brain tissues invasion [reviewed in [1]]. Even if the employment of highly active antiretroviral therapy (HAART) has changed the scenario of the HIV dementia by improving the cognitive performance in some patients with HIV-associated cognitive impairment, new cases of HIV dementia continue to develop [3]. In addition, the prevalence of HIV dementia is rising as patients on HAART live longer with HIV infection [3] suggesting that, in the era of HAART, HAD or milder forms still remain high [4]. Thus, the neuropathogenesis of HIV-infection and better therapeutic approaches for the management of neuroAIDS still remain to be elucidated.

Evidence exists that astrocytes may play a role in HIV-related neurological disorders. Indeed, reactive astrogliosis and the presence of activated and hypertrophied astrocytes, has commonly been described in the brain of patients suffering from HAD [5,6]. On the other hand, in vivo studies have also shown that HIV infection mat also occur in a small and variable fraction of astrocytes, mainly in advanced brain disease [7-11], thus suggesting that astrocyte are actively involved in the pathogenesis of HAD.

Although the incidence of astroglial cell death in HIV-infected brain tissues is still to be better clarified, evidence suggests that astrocytes may also suffer dysfunction in the HIV-infected brain that may extend beyond the limited levels of HIV- infection and contribute to neuropathogenesis in distinct pathways [reviewed in [12-15]]. It is known that astrocytes are likely exposed continuously to HIV particles, viral proteins, cytokines, and other substances secreted by HIV-infected macrophages and microglia. Although they lack CD4 they express CXCR4, and under certain circumstances, CCR3 and CCR5, the co-receptors for HIV entry into cells [reviewed in [16-18]]. These chemokine receptors can transduce responses to chemokines and to HIV gp120 present in the brain and they might be involved in HIV association with astrocytes. Studies *in vitro *indicate that many of these products significantly modulate astrocyte physiology which in turn can alter essential interactions of astrocytes with other cells in the brain, particularly neurons. For example, exposure of cultured astrocytes to HIV, recombinant gp120, or viral transactivator Tat induces some of the same secretable mediators of neuropathogenesis as those produced by macrophages, including inflammatory cytokines TNF-α and IL-1β, chemokines MCP-1 and IP-10, IL-6, or free radicals such as nitric oxide and peroxynitrite [19-29].

The apparent dysregulation of astrocyte immune functions might contribute to the overall inflammatory environment in the brain but also may account for excitotoxic mechanisms which have been shown to occur in HIV-infected brain tissues. In particular, evidence exists that glutammatergic transmission is inbalanced in the brain of HAD patients. In particular, studies in culture revealed that intact HIV or exogenous gp120-induced extensive changes in astrocyte gene expression [30-33] and impaired transport of extracellular glutamate by astrocytes [34,35], a defect which may lead to neuronal death by glutamate excitotoxicity [36]. Glutamate uptake can also be impaired by intracellular expression of recombinant Tat or exposure of astrocytes to TNF-α [37]. On the other hand, intracellular Ca^2+ ^is increased by gp120 in neurons and astrocytes, an effect driven by enhanced glutamate receptor signalling [38]. Finally, recombinant gp120 was shown to induce Ca^2+^-dependent glutamate secretion by astrocytes and neuronal cell death in glial-neuronal co-cultures in a pathway involving signaling through CXCR4 and production of TNF-α [39,40]. Thus, HIV-related neurotoxins, such as gp120, might disregulate astrocytic viability via multiple mechanisms including an imbalanced modulation of glutamate turnover.

The present study has been performed to investigate 1) the effect of N-acetylcysteine (NAC), on gp120-induced changes of viability of human cultured astroglial cells and its correlation with gp120-related changes of lipid peroxidation ; 2) the effect of NAC on gp120-induced changes of glutamine synthase (GS), the enzyme which metabolises glutamate into glutamine in astroglial cells thereby playing a neuroprotective role [41].

## Results

**Figure 1:**
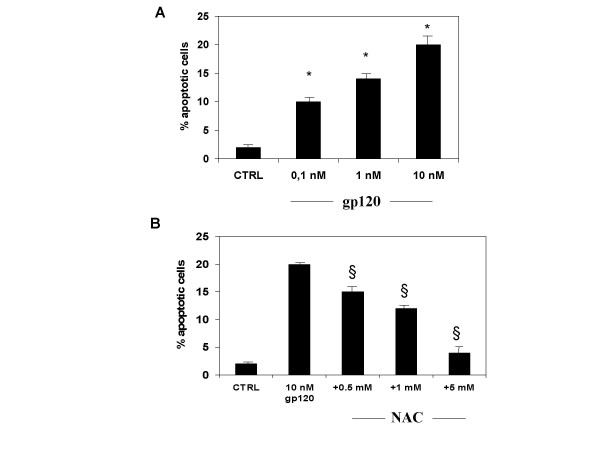
**N-acetylcysteine (NAC) prevents gp120-induced apoptotic cell death of cultured astroglial cells**. A. Incubation of astroglial cells with gp 120 (0.1,1 and 10 nM) for 24 hours reduced cell viability compared with untreated controls, as assessed by FACS analysis. B. NAC (0.5–5 mM), in pre-treatment of two hours, prevented this effect. Data represent the mean ± S.E.M. of five independent experiments.* P < 0.05 gp 120-treated astrocytes vs control (untreated) cells; § P < 0.05 NAC vs gp 120-treated cells. Statistical analysis was done using ANOVA followed by Student-Newman-Keuls test.

**Figure 2:**
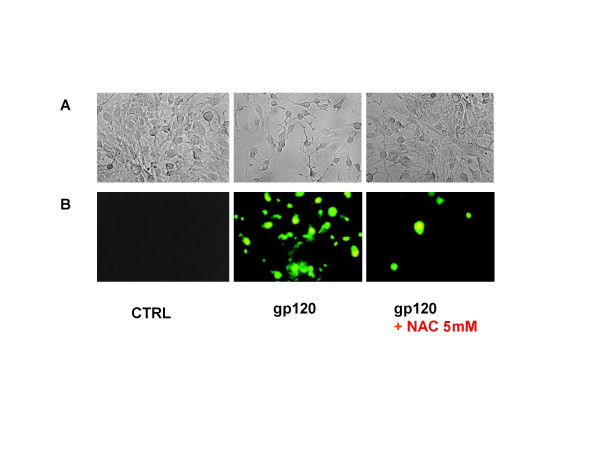
**NAC prevents apoptotic cell death of cultured astroglial cells induced by gp 120**. Incubation of astroglial cells with gp 120 (10 nm) for 24 hours leads to reduced cell population (A: phase contrast microphotographs) and DNA fragmentation as shown by appearance in preparation of TUNEL-positive cells (green) (B: immunofluorescence microphotographs) compared with untreated control. Pre-incubation of 2 hours with NAC (5 mM) antagonized the generation of apoptosis in astrocytes subsequent to incubation with gp 120 (10 nM). These are representative photomicrographs out of five independent experiment.

Incubation of human cultured astroglial cells with gp 120 (0.1–1 and 10 nM; n = 10 for each dose) for 24 h produced a decrease of cell viability as shown by counts of pre-treated astrocytes performed in trypan blue (Table 1). The reduction of astroglial cell viability due to incubation with gp120 was accompanied by apoptotic cell death. Indeed, measurement of apoptotic cell death as quantified by means of FACS analysis showed an enhanced number of hypoploid cells indicating programmed cell death due to gp120 incubation (Figure 1A; n = 5 for each dose). This was confirmed by immunocytochemical assay by means of Tunel reaction which showed apoptotic nuclei and DNA fragmentation (Figure 2; n = 5 for each dose).

**Table 1:** Gp120 reduced astroglial cell viability as expressed by % trypan blue positive cell counts in 10 experiments. NAC (0.5–5 mM) antagonized this effect.

**TREATMENT**	**% dead cells**
CTRL	2 ± 1.3
Gp120 100 pM	16 ± 1.4*
Gp120 1 nM	38 ± 2*
Gp120 10 nM	45 ± 3.2*
Gp120 10 nM + NAC 0.5 mM	40 ± 2.4§
Gp120 10 nM + NAC 1 mM	22 ± 2.1§
Gp120 10 nM + NAC 5 mM	6 ± 2§

P < 0.05 gp120-treated astrocytes vs control (CTRL)

§ P < 0.05 NAC vs gp120-treated astrocytes

**Figure 3:**
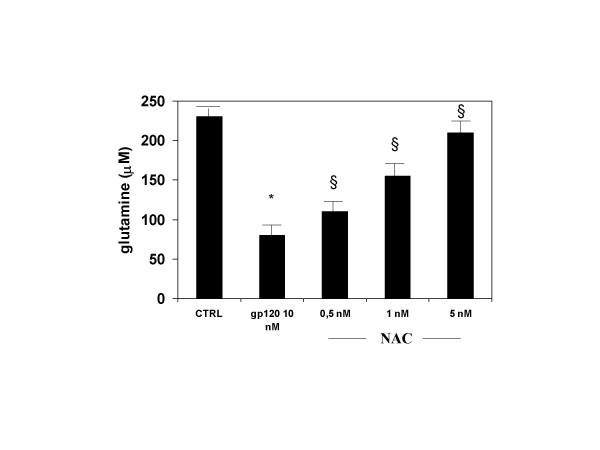
**The decrease of glutamine formation in the supernatant of astroglial cells incubated with gp 120 is reversed by NAC**. Treatment of astroglial cells with gp 120 (10 nM) for 24 hours produced a reduction of glutamine formation compared with untreated control. NAC (0.5–1 and 5 mM) in pre-treatment of two hours, reversed this effect. Data represent the mean ± S.E.M. of five independent experiments.* P < 0.05 when compared to control; § P < 0.05 treated vs gp 120 treated cells. Statistical analysis was done using ANOVA followed by Student-Newman-Keuls test.

**Figure 4:**
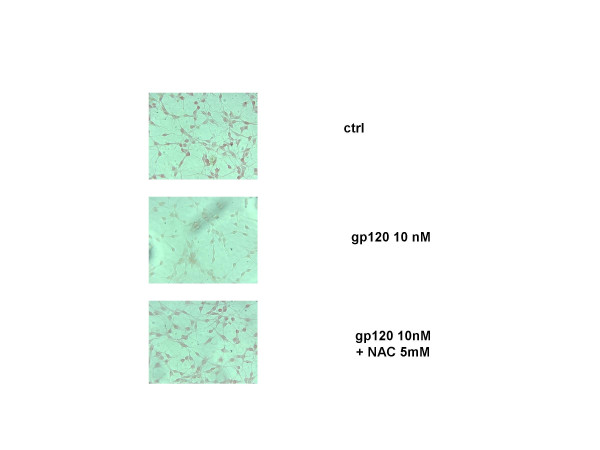
**NAC restores gp 120-related decrease of GS expression**. Glutamine synthase staining in astroglial cells either untreated or treated with gp 120 (10 nM) and gp 120 plus NAC (5 mM) for 24 hours. In particular, gp 120 reduced the immunocytochemical expression of glutamine synthase, while pre-incubation of cells with NAC restores gp 120- related decrease of GS in astrocytes. These are representative photomicrographs out of five independent experiments.

**Figure 5:**
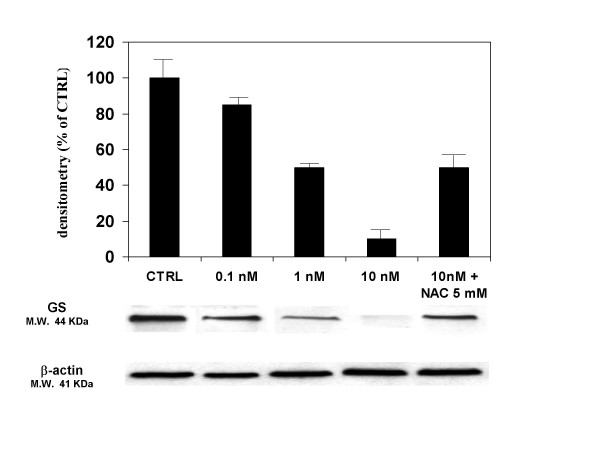
**The effect of gp120 on glutamine synthase expression in human cultured astroglial cells**. Incubation of astroglial cells with gp 120 (0.1, 1, 10 nM) for 24 hours dose-dependently reduced the GS expression as measured by western blotting analysis. Pre-treatment of 2 hours with NAC (5 mM) antagonized this effect. Blots are representative of 5 experiments. The columns represent the mean ± S.E.M %

The effect of gp 120 on cell viability was accompanied by a reduction of glutamine formation and GS expression in gp 120-treated astroglial cells. Indeed, incubation of cultured astroglial cells with gp 120 (10 nM; n = 5) for 24 h, produced a significant decrease of the amount of glutamine in cell supernatants as measured by glutamine assay (Figure 3). These results were confirmed by western blotting analysis and immunocytochemistry which showed that gp 120 (10 nM; n = 5) reduced significantly GS expression in gp 120-treated astroglial cells (Figures 4 and 5).

**Figure 6:**
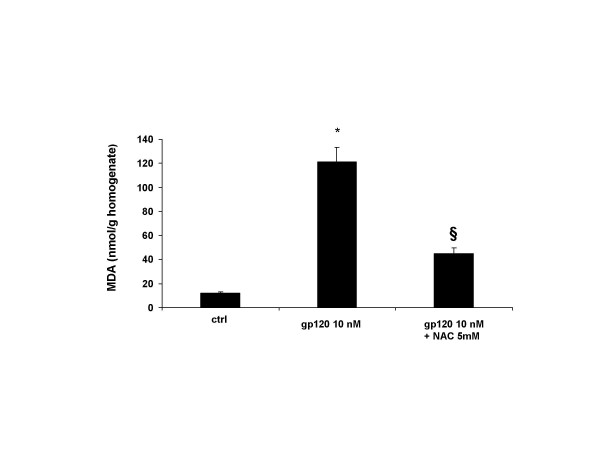
**The elevation of malondihaldehyde (MDA) levels in astroglial cell homogenates induced by gp120 is antagonised by NAC**. MDA increased within astroglial cells incubated with gp 120 (10 nM) for 24 hours. Pre-treatment of 2 hours with NAC (5 mM) antagonized MDA overproduction. Data represent the mean ± S.E.M. of five independent experiments.* P < 0.05 when compared to control; § P < 0.05 treated vs gp 120 treated cells. Statistical analysis was done using ANOVA followed by Student-Newman-Keuls test.

The effect of gp 120 was associated with overproduction of free radical species and subsequent lipid peroxidation. Indeed, incubation of cultured astrocytes with gp 120 (10 nM; Figure 6; n = 5), produced a prominent elevation of MDA levels in cell homogenates thus suggesting that the astroglial cell damaging due to incubation with gp 120 on astrocytes was accompanied by oxidative stress.

Pre-incubation of astroglial cells cells with NAC (0.5–1 and 5 mM; n = 10 for each dose; Table 1), restored astroglial cell viability in gp 120-treated cells. In addition, NAC dose-dependently antagonized the pro-apoptotic effect of 10 nM of gp 120 when quantified by FACS analysis (Figure 1B), an effect confirmed by TUNEL immunostaining when higher concentration of NAC (5 mM; n = 5; Figure 2) was used. In addition, NAC (0.5–1 and 5 nM; n = 5 for each dose; Figure 3) restored the concentration of glutamine in cell supernatant in gp120-treated astrocytes (10 nM), and the GS expression as evaluated by means of western blotting analysis (n = 5; Figure 5) an effect confirmed by GS immunostaining in gp 120-treated cells (gp 10 nM vs NAC 5 mM; n = 5; Figure 4). Finally, NAC (5 mM) inhibited MDA accumulation due to gp 120 (10 nM; n = 5; Figure 6) thus suggesting that antioxidant properties of NAC might contribute in restoring the imbalanced GS activity subsequent to incubation of astrocytes with gp 120.

## Discussion

The present data show that gp 120, a coating component of HIV envelope which has been shown to possess neurotoxic properties [42-45], leads to apoptotic cell death of human cultured astrocytes, an effect antagonised by NAC. This effect seems, at least in part, to be related to the antioxidant effect of this compound even though other effects of NAC on pathophysiological events leading to gp120-related astroglial cell injury cannot be excluded. The effect of NAC on gp120-induced reduction of astroglial cell viability is in accordance with previous data from our [29,46] and other groups [reviewed in [47]] showing that oxidative stress may play a crucial role in the pathogenesis of HAD. Our data also show, for the first time, that gp120 leads to an imbalanced GS activity and expression in cultured astrocytes this effect being accompanied by abnormal generation of free radicals and inhibited by NAC. This suggests that enhanced glutamatergic neurotransmission, which has been shown to contribute in HIV-related neurological disorders, may involve oxidative-mediated changes of GS in astrocytes.

Evidence exists that glutamate is highly neurotoxic when accumulated in massive amount in the extracellular space [48,49]. In the CNS, the conversion of glutamate to glutamine, that takes place within the astrocytes, represents a key mechanism in the regulation of excitatory neurotransmission under normal conditions as well as in injured brain [50]. In particular, it is known that the synaptically released glutamate is taken up by astroglial cells and then converted into nontoxic glutamine by the glia-specific enzyme glutamine synthase (GS); on the other hand, glutamine re-enters the glutamatergic neuron where is converted by glutaminase into glutamate, thus replenishing the neurotransmitter pool [51,52]. During pathological conditions such as HAD, stroke or several chronic neurodegenerative diseases, the duration and intensity of glutamate release might overwhelm the capacity of the GS enzyme to remove it. Such an imbalance should result in glutamate-mediated neurotoxicity and could contribute to brain injury supporting the hypothesis that an increase in GS expression exerts a neuroprotective effect [51]. The possible modulation of GS activity has been studied during numerous neuropathological states, including inflammation, ischemia/reperfusion injury etc. [50]. In particular, overproduction of reactive oxygen species, which occurs during excitotoxicity in brain tissues, leads to reduced ability of astroglial cells to regulate glutamate turnover via inhibition of GS activity [50-52]. In addition, oxidative inactivation of GS during ischemia/reperfusion in the gerbil's brain has been described indicating that free radical-dependent inactivation may be a critical factor in the neurotoxicity which follows ischemia/reperfusion injury [50]. On the other hand, it has been suggested that peroxynitrite, generated by the reaction between nitric oxide (NO) and superoxide anions [53], leads to nitration of GS tyrosine residues thus leading to significant dysregulation of proteins undergoing adenylylation regulation in signal transduction cascades [54-57]. Thus, the dysfunction of glutamate metabolism, which has been suggested to contribute in HIV-related neuropathogenesis, might occur also via the contribution of gp 120-mediated oxidative stress, an effect mediated by glutamine metabolism dysfuntion which, in turn, might contributes in apoptotic cell death of brain cells which follows HIV infection of brain macrophages.

## Conclusion

In conclusion, the present experiments demonstrate that gp 120 is neurotoxic in astroglial cells, an effect accompanied by lipid peroxidation and by altered glutamine release. All the effect of gp120 on astroglial cells were counteracted by NAC thus suggesting a novel and potentially useful approach in the treatment of glutamatergic disorders found in HAD patients.

## Methods

### Astrocyte cultures

The astrocytic cell line Lipari was derived from a 51 year old male patient who presented a large right front-temporal mass (astrocytoma), and grown as previously described [29]. Cells were expanded and cultured by seeding them in 25 cm^2 ^plastic flasks at a density of 0.7 × 10^6 ^cells/flask in DMEM supplemented with 10% foetal calf serum, 2 mM L-glutamine, 1 mM sodium pyruvate, 100 units/ml penicillin, 100 μg/ml streptomycin (complete medium), and incubated at 37°C in humidified air containing 5% CO_2_. To assess astroglial cell viability in cells either untreated or pre-treated with gp120, counts in trypan blue were performed.

### Gp120

HIV envelope gp120 glycoprotein was used all over the present experiments. In particular, recombinant HIV-1 (IIIB) full length and glycosylated gp120 with a molecular weight of approx 115 kda, was produced using the baculovirus expression system (purity: > 90% by SDS-PAGE). Gp 120 was purchased from RDI Division of Fitzgerald Industries Intl, Concord, MA, USA. To assess whether or not the effect of gp120 might be not due to endotoxin contamination, some experiments were performed by adding Polymyxin B (10 μg/ml ; n = 5 not shown), showing that gp120 was endotoxin-free.

### Flow cytometric analysis (FACS)

Astroglial cells either exposed or not to gp120 were trypsinised and then gently detached from plastic 6–8 days after exposure. Aliquots of 5 × 10^5 ^cells were centrifuged at 300 g for 5 min; pellets were washed with PBS, placed on ice, and overlaid with 0.5 ml of a hypotonic fluorochrome solution containing 50 μg/ml propidium iodide, 0.1% sodium citrate, and 0.1% Triton X-100. After gentle resuspension in this solution, cells were left at 4°C for 30 min., in absence of light, before analysis. Propidium iodide-stained hypoploid cells were analysed with a FACScan Flow Cytometer; fluorescence was measured between 565 and 605 nm. The data were acquired and analysed by the Lysis II program.

### TUNEL reaction

Astroglial apoptotic nuclei produced after incubation of astrocytes with gp 120 were assessed by *in situ *terminal deoxynucleotidyl transferase (TdT)-mediated dUTP-biotin nick-end labelling (TUNEL) of DNA strand breaks. Briefly, astroglial cells were permeabilized by a 20 min. incubation at room temperature in 0.15 Triton X 100/0.15 sodium citrate (% w/v). Following permeabilization, the slides were washed with PBS and the TUNEL reaction was done using the fluorescein in situ cell death detection kit (Roche, Germany). Immunofluorescence microscopy was performed on an epifluorescence microscope equipped with narrow bandpass excitation filters mounted in a filter wheel (Ludl Electronic Products, Hawthorne, NY). Images were captured by using a chilled, cooled charge-coupled device camera (Photometrics, Tucson, AZ) and Smart Capture software (Digital Scientific, Cambridge, UK) on a Macintosh computer.

### Glutamine assay

Glutamine changes in supernatants of human astroglial cells was determined by using a glutamine assay kit (Sigma) based on the reductive deamination of glutamine by a proprietary enzyme. The reaction is specific for glutamine and does not cross-react with other amino acids or ammonia. Briefly, cell supernatants, glutamine standards and cell culture medium were incubated with the reaction buffer, the diluent buffer and the specific enzyme for 1.30 h at 37°C. The colour reagent was added to each sample and stand for 5–10 min at room temperature. Absorbance was measured at 550 nm using a spectrophotometer. To calculate the quantity of glutamine a linear regression analysis of the standard curve was performed.

### Western blotting analysis

GS expression has been evaluated by means of western blotting analysis [19]. In particular, astroglial cell monolayers cultured either untreated or pre-treated with gp 120 alone or gp 120 plus NAC were washed three times with PBS and solubilized by direct addition of a preheated (to 80°C) denaturing buffer, containing 50 mM Tris-Cl pH 6.8, 2% SDS and protease inhibitor cocktail (Sigma). Solubilized samples were collected and immediately boiled for 2 min. Bromophenol blue, glycerol and β-mercaptoethanol were then added to final concentrations of 0.05%, 10% and 2%, respectively. Samples were boiled again before loading onto SDS-PA gels. After electrophoresis, polypeptides were electrophoretically transferred to nitrocellulose filters (BioRad). Monoclonal anti-Glutamine Synthase (1:3000, Transduction Laboratories) and monoclonal anti-β-actin (1:5000, Sigma) antibodies were used to reveal the respective antigens. After incubation with secondary reagent (1:5000, anti-mouse horseradish peroxidase conjugate from Transduction Laboratories), blots were developed with the enhanced chemiluminescense procedure, using reagents (ECL-Plus) from Amersham Life Science (Buckinghamshire, UK).

### Immunocytochemistry

Cells grown on glass coverslips were briefly rinsed with phosphate buffered saline (PBS, pH 7.4) and fixed with 4% paraformaldehyde in 0.120 M sodium phosphate, pH 7.4, for 30 min at 37°C. Cells were then rinsed in PBS, incubated with 0.01% H_2_O_2 _in methanol to inactivate endogenous peroxidases, permeabilized in 0.5% triton X-100 for 5 min, and rinsed twice with PBS. Coverslips were inverted onto droplets of 5% horse serum in 1% bovine serum albumine (BSA) in PBS for 1 h at room temperature. The cells were then rinsed in PBS and incubated with primary monoclonal antibody against the glutamine synthase (Transduction Laboratories) diluted 1:500 in 1% BSA/PBS, overnight at 4°C. The cells were washed in PBS, incubated with the biotinylated secondary antibody and peroxidase-conjugated streptavidin each for 30 min at room temperature, and then rinsed in PBS. Peroxidase activity was revealed using 3,3'-diaminobenzidine-HCl. Coverslips were mounted in Vectashield and viewed under a light microscope.

### Malondialdehyde Determinations

Malondialdehyde (MDA), used as a biochemical marker for lipid peroxidation, was measured by a method previously described [42]. Levels of MDA were measured 3–12 h after gp 120 incubation with astroglial cells. Briefly, cells were homogenized in potassium chloride (1.15%) and frozen in liquid nitrogen. Chloroform (2 ml) was then added to each homogenate and then spun for 30 min.

The organic layer of the sample was removed and dried under nitrogen gas and re-constituted with 100 μl of saline. MDA generation was evaluated by the assay of thiobarbituric acid (TBA)-reacting compounds. The addition of a solution of 20 μl of sodium dodecyl sulphate (SDS; 8.1%), 150 μl of 20% acetic acid solution (pH3.5), 150 μl of 0.8% TBA and 400 μl of distilled water, produced a chromogenic product which was extracted in n-butanol and pyridine. Then, the organic layer was removed and MDA levels read at 532 nm and expressed as nmol MDA/g prot.

### Statistical analysis

Results are given as mean ± sem. Statistical analysis was performed using ANOVA followed by Student- Newman-Keuls. P < 0.05 was considered statistically significant.

All the experiments have been carried out according to the ethical and consent approval guidelines stated by Italian Ministry of Research.

## Authors' contributions

VV , CM and CC carried out cell cultures, the molecular biology studies and drafted the manuscript. EP, FS and IS carried out the immunoassays. DR and NC participated in the design of the study and performed the statistical analysis. VM conceived of the study, and participated in its design and coordination. All authors read and approved the final manuscript.
